# Immunogenic chemotherapy: great potential for improving response rates

**DOI:** 10.3389/fonc.2023.1308681

**Published:** 2023-12-06

**Authors:** Xiaojun Huang, Qinghuan Ren, Leixiang Yang, Di Cui, Chenyang Ma, Yueliang Zheng, Junjie Wu

**Affiliations:** ^1^ Cancer Center, Department of Pulmonary and Critical Care Medicine, Zhejiang Provincial People’s Hospital (Affiliated People’s Hospital), Hangzhou Medical College, Hangzhou, Zhejiang, China; ^2^ Alberta Institute, Wenzhou Medical University, Wenzhou, Zhejiang, China; ^3^ Cancer Center, The Key Laboratory of Tumor Molecular Diagnosis and Individualized Medicine of Zhejiang Province, Center for Reproductive Medicine, Department of Genetic and Genomic Medicine, Zhejiang Provincial People’s Hospital (Affiliated People’s Hospital), Hangzhou Medical College, Hangzhou, Zhejiang, China; ^4^ Cancer Center, The Key Laboratory of Tumor Molecular Diagnosis and Individualized Medicine of Zhejiang Province, Zhejiang Provincial People’s Hospital (Affiliated People’s Hospital), Hangzhou Medical College, Hangzhou, Zhejiang, China; ^5^ Department of Internal Medicine of Traditional Chinese Medicine, The Second People’s Hospital of Xiaoshan District, Hangzhou, Zhejiang, China; ^6^ Cancer Center, Emergency and Critical Care Center, Department of Emergency Medicine, Zhejiang Provincial People’s Hospital (Affiliated People’s Hospital), Hangzhou Medical College, Hangzhou, Zhejiang, China

**Keywords:** chemotherapy, immunogenic cell death, immunotherapy, anti-tumor immune response, dose and schedule, biomarker

## Abstract

The activation of anti-tumor immunity is critical in treating cancers. Recent studies indicate that several chemotherapy agents can stimulate anti-tumor immunity by inducing immunogenic cell death and durably eradicate tumors. This suggests that immunogenic chemotherapy holds great potential for improving response rates. However, chemotherapy in practice has only had limited success in inducing long-term survival or cure of cancers when used either alone or in combination with immunotherapy. We think that this is because the importance of dose, schedule, and tumor model dependence of chemotherapy-activated anti-tumor immunity is under-appreciated. Here, we review immune modulation function of representative chemotherapy agents and propose a model of immunogenic chemotherapy-induced long-lasting responses that rely on synergetic interaction between killing tumor cells and inducing anti-tumor immunity. We comb through several chemotherapy treatment schedules, and identify the needs for chemotherapy dose and schedule optimization and combination therapy with immunotherapy when chemotherapy dosage or immune responsiveness is too low. We further review tumor cell intrinsic factors that affect the optimal chemotherapy dose and schedule. Lastly, we review the biomarkers indicating responsiveness to chemotherapy and/or immunotherapy treatments. A deep understanding of how chemotherapy activates anti-tumor immunity and how to monitor its responsiveness can lead to the development of more effective chemotherapy or chemo-immunotherapy, thereby improving the efficacy of cancer treatment.

## Introduction

Conventional chemotherapy agents are relatively inexpensive and widely used in treating cancers in clinics. Clinicians usually have extensive knowledge of the toxicity profiles associated with chemotherapy due to its long history of usage. Therefore, it has special merits to make the best use of chemotherapy, especially for cancer patients in developing country where there is limited access to state-of-the-art therapies such as surgery, radiation therapy, targeted chemotherapy or immunotherapy. Conventional chemotherapy aims to kill or slow the growth of cancer cells. However, the overall efficacy of chemotherapy is limited, while great achievements have been made in the past decades (1, 2).

The activation of endogenous anti-tumor immunity is critical in treating cancers (3–5). This has been firmly validated by the use of immune checkpoint inhibitors (ICI)-based immunotherapy, which have made significant progress in treating cancers over the past few years (6, 7). Some clinical trials also found that the efficacy of chemotherapy is correlated with the level of anti-tumor immune responses (3, 5). In addition, recent studies indicated that some chemotherapy agents, such as doxorubicin, mitoxantrone, epirubicin, idarubicin, oxaliplatin, and cyclophosphamide can stimulate anti-tumor immunity by inducing immunogenic cell death (ICD) of tumor cells (8, 9). Furthermore, chemotherapy can induce complete tumor regression through a mechanism of activating anti-tumor immunity in preclinical studies (10, 11). These studies suggested that immunogenic chemotherapy holds great potential to improve the efficacy of chemotherapy and immunotherapy.

However, in practice, most standard-of-care (SOC) chemotherapy agents are still administrated on a maximum tolerated dose (MTD) schedule that is immune suppressive. Regarding the chemo-immunotherapy regimens that combine chemotherapy with immunotherapy, it is rarely studied whether the dose and schedule used is optimal for activating anti-tumor immune responses. Consequently, the overall rate of long-term survival or cure of chemo-immunotherapy is still limited, even though numerically significant improvements in parameters such as pathological complete responses and progress-free survival have led to their approval in treating multiple types of cancers (12–15).

Then, why is not chemotherapy used in clinics focused on their immune activation function? We think that it is because the dose and schedule dependence of chemotherapy on activating anti-tumor immune response has not been fully appreciated. Furthermore, the optimal dose and schedule of chemotherapy may vary depending on the type and stage of cancer and is difficult to achieve without careful optimization (11, 16–18). Moreover, combination therapy may be required when the cytotoxicity against tumor cells is insufficient due to chemotherapy protocol or when the immune responsiveness is low due to intrinsic features of the tumor model (11, 18–20). Understanding and addressing these problems may help design better chemotherapy or chemo-immunotherapy treatment and improve the efficacy of cancer therapy.

## A model of immunogenic chemotherapy treatment-induced long-lasting response

**Figure 1:**
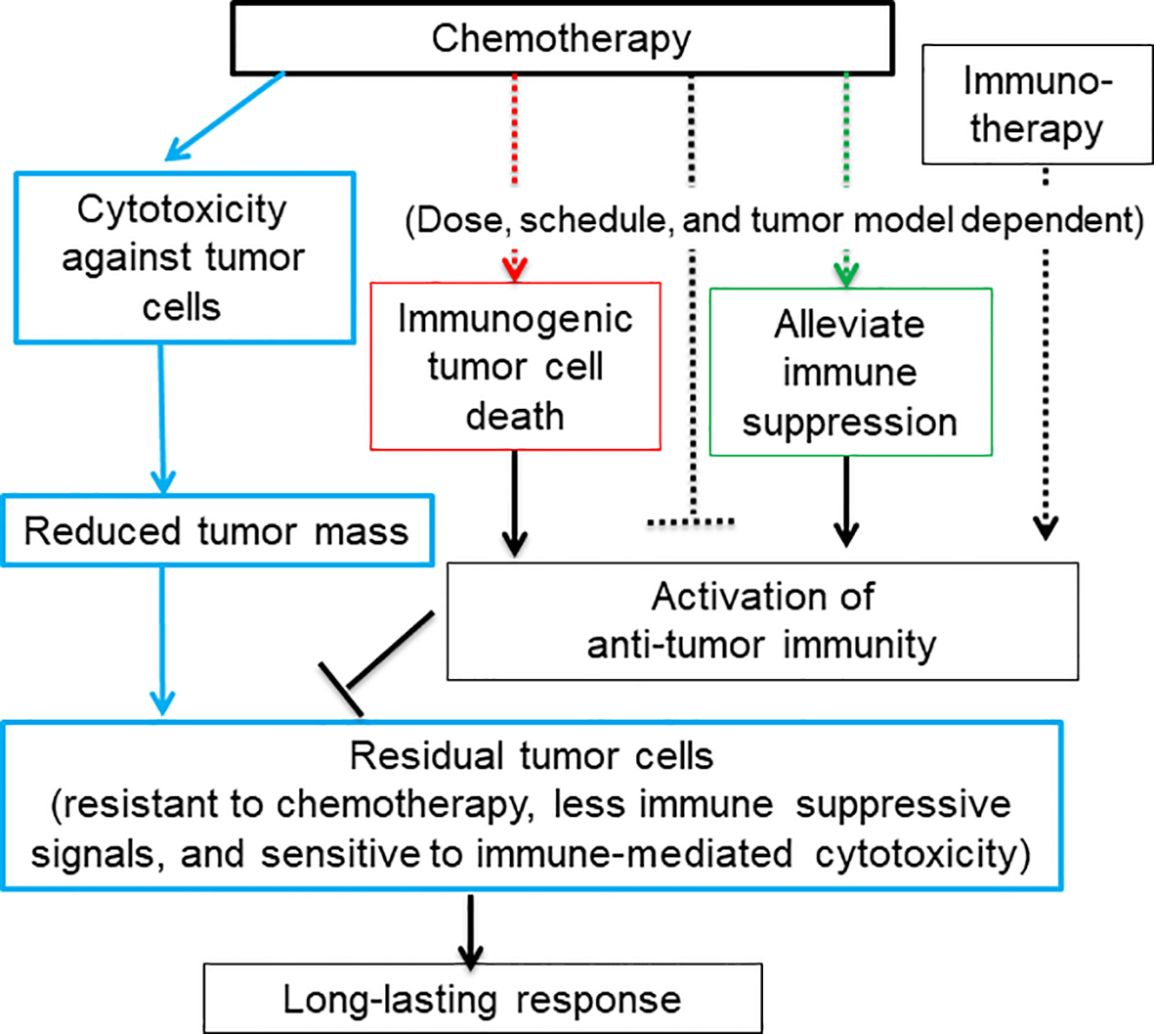
A diagram depicts that the dual function of chemotherapy in killing tumor cells and activating anti-tumor immune responses may result in a long-lasting response. Blue rectangular and arrow: Chemotherapy kill or inhibit tumor cell growth via cytotoxicity against tumor cells, which greatly reduces tumor mass and tumor-cell derived immune suppressive signals. The rest of tumor cells that are resistant to chemotherapy may be sensitive to immune-mediated cytotoxicity. Some chemotherapy agents are capable of activating anti-tumor immune responses via inducing immunogenic tumor cell death (red rectangular and arrow) and eliminating or modulating immune suppressive cells and thus restoring anti-tumor immunity (green rectangular and arrow). Chemotherapy can also inhibit anti-tumor immune responses via lymphopenia effects (black dashed arrow). The activation of anti-tumor immunity is heavily dependent on the dose and schedule of chemotherapy. For low-immune responsiveness tumor models or low-dosage chemotherapy, chemotherapy in combination with immunotherapy may be required to achieve a long-lasting response.

The chemotherapy-induced ICD starts with a tumor cell intrinsic damage-associated molecular pattern (DAMP), including translocation of calreticulin, an endoplasmic reticulum chaperone protein, to the tumor cell surface (21), and release of lysosomal ATP (22), nuclear chromatin binding protein HMGB1 (23, 24), cytosolic protein annexin A1 (25), and/or various nucleic acids (16, 17, 24, 26, 27). Together with immune-stimulating cytokines and chemokines secreted from stressed host cells and tumor cells (26, 28, 29), DAMPs trigger specific pattern recognition receptors (PRRs) on myeloid cells (9, 30), which further activate T cells to mediate anti-tumor immune responses (**Figure 1**, red rectangular and arrow).

We have reviewed immune modulation function of nine representative commonly used chemotherapy agents including cyclophosphamide, doxorubicin, mitoxantrone, cisplatin, oxaliplatin, gemcitabine, 5-fluorouracil, docetaxel, and paclitaxel (**Table 1**). Besides of four well-known immunogenic chemotherapy agents, i.e., cyclophosphamide, doxorubicin, mitoxantrone, and oxaliplatin (21, 68), all other agents are able to stimulate maturation of dendritic cells and activate anti-tumor activity of T cells. This suggests that most chemotherapy agents used in clinics now have potential to induce ICD of tumor cells, even though some chemotherapy agents have not been studied using the classical vaccine validation assay (9, 15).

**Table 1:** The Differential effects of chemotherapy treatment on immune cell modulation.

Chemotherapy drugs	Treg cells	T cells	Dendritic cells	Macrophages	MDSCs
Cyclophosphamide	Depletion (10, 31)	Depletion followed by activation (10)	Maturation (32)	Re-polarize to M1-like (33, 34)	Re-polarize to M1-like or depletion (34)
Doxorubicin	Depletion (35)	Activation (26, 36)	Maturation (26, 37, 38)	Re-polarize to M1-like (33)	Depletion (39)
Mitoxantrone	Depletion (41)	Depletion followed by activation (40)	Maturation (37, 38)	Inhibition (42)	Depletion (by pegylated liposomal formulation) (41)
Cisplatin	Depletion (43)	Activation (36, 44)	Maturation (45)	Re-polarize to M2-like (46); Be induced to present antigen (47)	Depletion (45)
Oxaliplatin	Depletion (48, 49)	Activation (44)	Maturation (44, 50)	Re-polarize to M1-like (51); Activated to recruit T cells (52, 53)	Depletion or re-polarize to M1-like depending on doses (48)
Gemcitabine	Depletion (54)	Activation (55)	Maturation (56)	Re-polarize to M2-like (57, 58)	Depletion (55, 59)
5-fluorouracil	Depletion (60)	Activation (59)	Maturation (50)	Re-polarize to M1-like (61)	Depletion (59)
Docetaxel	Inhibition (62)	Depletion followed by activation (62)	Maturation (37)	Re-polarize to M1-like (63)	Depletion; Re-polarize to M1-like (64)
Paclitaxel	Inhibition (65)	Activation (66)	Maturation (37, 38, 66)	Re-polarize to M2-like (67)	Re-polarize to M1-like (64)

Some chemotherapy agents can also restore anti-tumor immune responses through alleviating immune suppression (**Figure 1**, green rectangular and arrow) (**Table 1**). For example, immune suppressive regulatory T (Treg) cells can be either efficiently inhibited by paclitaxel and docetaxel or depleted by many other chemotherapy agents including cyclophosphamide., respectively (see **Table 1** for more references) (69). Myeloid-derived suppressor cells (MDSCs) can be depleted by docetaxel, gemcitabine, cisplatin, cyclophosphamide, oxaliplatin, docetaxel and 5-flurouracil (64, 70). MDSCs can also be re-polarized into anti-tumor M1-like status by cyclophosphamide, oxaliplatin, docetaxel, and paclitaxel in different tumor models or study conditions. Pro-tumor M2-like macrophages can be re-polarized into anti-tumor M1-like by doxorubicin, oxaliplatin, 5-fluorouracil, mitoxantrone, docetaxel and cyclophosphamide (71).

In addition, chemotherapy treatment increases the expression level of tumor neo-antigen and MHC-I molecules on the surface of tumor cells (4). These signals render residual tumor cells sensitive to the immune cell cytotoxicity. In the meanwhile, chemotherapy reduces or limits tumor volume through cytotoxicity against tumor cells. This helps reduce the immune suppressive signal arising from tumor cells (72) (**Figure 1**, blue rectangular and arrow) (4, 73). Thus, chemotherapy-activated anti-tumor immunity relay the killing of the remaining tumor cells after the initial wave of tumor cell killing mediated by chemotherapy-associated cytotoxicity. Since immunity and chemotherapy use different mechanisms to target tumor cells, the chance for tumor cells to evolve and select mutation to resist both is greatly reduced. Therefore, it is reasonable to hypothesize that immunogenic chemotherapy has great potential to improve the efficacy of chemotherapy or chemo-immunotherapy by inducing long-lasting responses through the induction of anti-tumor immunity (**Figure 1**) (4).

However, chemotherapy could be immune suppressive due to its lymphopenia effects (**Figure 1**, black dashed inhibitory arrow), which is one of common side effects associated with chemotherapy treatment. For example, depletion of T cells was seen before activation by treatment of cyclophosphamide, mitoxantrone and docetaxel (**Table 1**). Furthermore, some chemotherapy agents including cisplatin, gemcitabine and paclitaxel can re-polarize macrophages to M2-like to promote tumor growth and reduce the efficacy of chemotherapy (**Table 1**). For these agents, tumor models with low levels of macrophages could be preferential treatment targets. Alternatively, the efficacy of these chemotherapy agents can be improved by immunotherapy agents targeting macrophages. While immunogenic chemotherapy-associated feature looks appealing, it is thus heavily dose, schedule, and tumor model dependent (11, 74), and combination therapy may be required to complement the low chemotherapy dosage or low immune responsiveness due to intrinsic tumor features as we will discuss more later (11, 18) (**Figure 1**). Only when chemotherapy cytotoxicity against tumors interacts with anti-tumor immunity synergistically, can long-lasting responses be achieved.

## Conventional chemotherapy dosed on a MTD schedule

**Figure 2:**
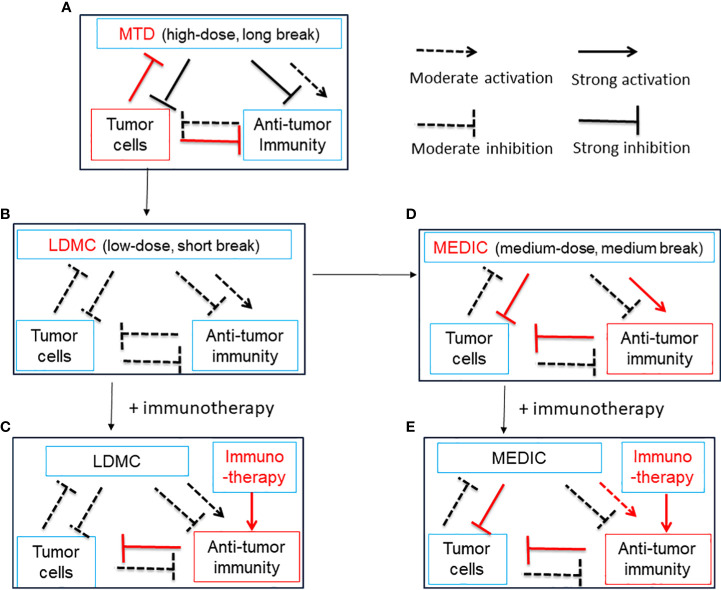
A simplified diagram illustrates the relationship between chemotherapy treatment, anti-tumor immunity and tumor cells. Chemotherapy can activate anti-tumor immunity by ICD of tumor cells and/or alleviation of immune suppression. Chemotherapy can also curtail anti-tumor immunity by lymphopenia effects. Anti-tumor immunity may kill tumor cells, and tumor cells may inhibit, evade and subvert anti-tumor immunity. Tumor cells may be killed by chemotherapy initially but develop resistance later during drug-free breaks. **(A)**, A model for MTD in which chemotherapy is administrated at a high-dose with long drug-free break. The MTD chemotherapy kills numerous tumor cells initially and activates transient anti-tumor immunity. Tumor cells develop significant drug resistance to chemotherapy and override anti-tumor immunity during long-drug free breaks, which is required for host cells to recover from high toxicity. Thus, there is a need to reduce the dosage of MTD chemotherapy. See **(B)** and **(D)** for solution. **(B)**, A model for LDMC in which the chemotherapy is administrated at a low-dose with short drug-free break. The LDMC exerts moderate cytotoxicity to tumor cells and encounters litter resistance from tumor cells. The LDMC may restore moderate anti-tumor immunity after depleting or modulating immune suppressive cells. Of note, due to lymphopenia effects of chemotherapy, the restoration of anti-tumor immunity usually occurs when LDMC is stopped or there is a relative long drug-free break. Overall, the moderate anti-tumor immunity associated with LDMC is not sufficient to durably regress tumors. See **(C, D)** for solution. **(C)**, A model showing LDMC in combination with immunotherapy. The immunotherapy may benefit from the improved tumor micro-environment set up by LDMC and thus the overall anti-tumor immunity could be greatly strengthened. **(D)**, A model for MEDIC in which chemotherapy is dosed at a medium-dose with medium drug-free break. Significant and sustained anti-tumor immunity could be activated attributed to ICD of tumor cells, alleviation of immune suppression, and optimal drug-free break that prevent immune exhaustion or immune suppression. The total dosage of MEDIC could be similar to MTD. The tumor cells suffered from double hits from chemotherapy and anti-tumor immunity have a low-chance to develop resistance and thus MEDIC may have high efficacy. **(E)**, A model showing MEDIC in combination with immunotherapy. Combining appropriated immunotherapy with MEDIC may help boost anti-tumor immunity and improve the cancer treatment efficacy. This is especially recommended for tumor models with low immune responsiveness to MEDIC treatment. The use of a dashed arrow or dashed inhibitory arrow denotes moderate activation or inhibition, while a solid arrow or inhibitory arrow denotes strong activation or inhibition. Red is used to draw attention to important items. ICD, immunogenic cell death; MTD, maximum tolerated dose; LDMC, low-dose metronomic chemotherapy ; MEDIC, medium-dose repeating intermittent chemotherapy.

Conventional standard-of-care (SOC) chemotherapy, aiming to eradicate all cancer cells, is dosed on a MTD schedule associated with severe toxicity to host cells, which necessitates a prolonged drug-free break to allow normal host cells to recover. The residual tumor cells will take advantage of this drug-free break to evolve and expand drug-resistant mutants, which reduce the efficacy of follow-up chemotherapy and culminate tumor relapse (75) (**Figure 2A**). However, this is a view based on a simplified two-element system only consisting of chemotherapy and tumor cells. In practice, the stromal cells, such as fibroblasts, endothelial cells, tumor-associated macrophages, and MDSCs, also contribute to tumor progression and resistance to chemotherapy treatment (76–78). For example, Chan et al. found that, for three commonly used standard chemotherapy agents, doxorubicin, paclitaxel, and cyclophosphamide, MTD regimens stimulate STAT-1 and NF-κB signaling in fibroblasts to express and secrete Glu-Leu-Arg (+) chemokines, which further convert and expand part of tumor cells into chemotherapy-resistant cancer stem cell-like cells (78).

In addition, immune suppressive signals developed during long drug-free break may override the anti-tumor immune responses and thus MTD schedule is not optimal for activating anti-tumor immune responses (32, 72) (**Figure 2A**). Preclinical studies from Doloff et al. indicated that, even immunogenic chemotherapy agent such as cyclophosphamide, MTD schedule only induced a transient anti-tumor immune response (32). Furthermore, MTD regimen-associated high toxicity often requires co-medication such as glucocorticoid (79), which is also immune suppressive. Therefore, considering the importance of anti-tumor immunity, there is a need to reduce the dosage of SOC chemotherapy and shorten the drug-free intervals. The differential immune response associated with MTD and other two chemotherapy treatment schedules (see below) is summarized in **Table 2**.

**Table 2:** The differential toxicity and immune responses associated with three different chemotherapy treatment schedules.

Chemo-therapy schedule	Dose	Lympho-penia	Myelo-ablation	Drug-free break	Cytotoxicity to tumor cells	Immune recovery/activation
LDMC	Low	Medium-strong	No	Short	Low	Moderate and transient*
MEDIC	Medium	Strong	No	Medium (6-7 days)	Medium-high#	Significant and persistent
MTD	High	Strong	Yes	Long (2-3 weeks)	High	Moderate and transient

*, In clinical setting, LDMC is sometimes carried out on a one-week on and one-week off schedule. Immune recovery may occur in the drug-free weeks. Alternatively, immune recovery may occur after LDMC treatment stops. However, if dosage of chemotherapy is so low that no lymphopneia effect is induced, immune recovery may occur during the LDMC treatment. #, The total dosage of MEDIC treatment could be high when medium doses are given every 6- or 7-day repeatedly. LDMC, low-dose metronomic chemotherapy ; MEDIC, medium-dose repeating intermittent chemotherapy; MTD, maximum tolerated dose.

Since mono-immunotherapy and mono-chemotherapy are often troubled by low responsiveness or drug tolerance, there is a great interest in studying the combination therapy encompassing (ICIs) and immunogenic chemotherapy (13, 80). However, in clinical trials that compare the efficacy of SOC chemotherapy with ICI plus chemotherapy, the same SOC chemotherapy drug and MTD schedule are often used in combination with immunotherapy (13, 81). Furthermore, the ICI and SOC chemotherapy are often administrated concomitantly due to the lack of knowledge that which sequence or schedule is more effective (13, 14). As a result, the SOC chemotherapy may deprive the targets of ICI due to strong lymphopenia and myelosuppression effects and thus reduce the efficacy of combination regimen. A time course study assaying the change of immune activation-related gene expression in response to chemotherapy or ICI treatment, respectively, may help optimize the dose and schedule and thus greatly improve the combinatory regimen efficacy, as we will discuss later.

## Overview of low-dose metronomic chemotherapy

Low-dose metronoic chemotherapy (LDMC) is a modified chemotherapy schedule characterized by low doses and short drug-free break or continuous administration (82) (**Table 2**) (**Figure 2B**). In contrast to MTD, LDMC is usually used as a palliative treatment for elderly and frail patients mainly because of relatively fewer toxicity-associated side effects and ease of administration (83, 84). Mechanistic study found that LDMC schedule switches the drug targets from tumor cells to tumor endothelial cells, and thus can potentially overcome the acquired tumor cell resistance to chemotherapy (82, 85). In contrast to bevacizumab that inhibits angiogenesis by blocking the action of angiogenesis factor VEGF (86), most LDMC regimens use different mechanisms by directly targeting endothelial cells or reducing the production of angiogenesis factors (87). For example, microtubule-targeting agent vinorelbine inhibits endothelial cell proliferation, migration, and sprouting (85). The LDMC topotecan can inhibit the translation and transcriptional activity of HIF-1α, a upstream regulator of angiogenesis effectors (88, 89). In addition, LDMC relieves stromal cell-mediated resistance to chemotherapy (78). For example, Chan et al. found that LDMC chemotherapy largely relieves STAT-1 and NF-κB paracrine signaling in fibroblasts to enhance the therapy effects (78).

Recent studies indicated that LDMC can also restore some immune function by eliminating immunosuppressive cells (90). Of note, sustained exposure to chemotherapy cytotoxicity may lead to lymphopenia, which can eliminate both the originally-available immune cells and the immune cells that could have been induced (11, 91). However, restoration of anti-tumor immunity may occur after LDMC treatment is stopped (90), or during one-week long drug-free break in some modified LDMC schedules as used by Ghiringhelli et al. (31), which is more like intermittent schedules as we will discuss later.

## Combination regimen to improve LDMC efficacy

A reasonable concern for LDMC is that low-level cytotoxicity will not be enough to limit overall tumor cell growth, which may eventually override immune responses. For example, expanded tumor mass may generate a micro-environment against anti-tumor immune cell infiltration, subvert anti-tumor immune cells, and/or expand tumor cells with low MHC-I molecules to evade immune surveillance (4, 74).

One way to circumvent the problems caused by low-dose chemotherapy is to have combination therapy. For example, doxorubicin or its nanoparticle formulation, Doxil, cannot significantly limit mammary tumor 4T1 and E0771 progression at low dosage (20). However, the efficacy of doxorubicin or Doxil could be greatly improved by tumor micro-environment normalization induced by repurposed tgfb inhibitor Transilst, an approved anti-fibrotic and anti-histamine drug (20). Furthermore, Doxil enhanced Transilst-mediated tumor blood vessel function, increased immunostimulatory M1 macrophage content and improved the efficacy of the ICI antibodies anti-PD-1/anti-CTLA-4 (**Figure 2C**) (20). Jordan et al. found that CpG-1826 (a TLR9 agonist) immunotherapy can significantly increase the efficacy of low- or moderate-dose cyclophosphamide (45 or 90 mg/kg) treatment by enhancing anti-tumor immune responses (**Figure 2C**) (19).

In clinical trials, LDMC is mostly used as a precondition regimen to set up a favorable micro-environment for immunotherapy such as oncolytic virus and tumor vaccines, which has been reviewed by Fabian et al. and Chen et al. (14, 92) (**Figure 2C**). In few cases, LDMC is used as induction regimen to potentiate ICI. For example, LDMC vinorelbine in combination with atezolizumab is studied as a second-line treatment for patients with stage IV non-small cell lung cancers (93). For similar reason, LDMC is also used in clinics as a combination regimen with conventional chemotherapy, targeted therapy and radiotherapy (94, 95). Although these combination therapies are expected to be more effective than mono-LDMC as a palliative therapy, they have not yet been shown to result in durable tumor regression or long-lasting responses (14, 92).

## Low- or moderate-dose chemotherapy has advantage in activating anti-tumor immunity

Different from LDMC restoring immunity by alleviating immune suppression, some chemotherapy agents can activate robust anti-tumor immune responses through inducing ICD of tumor cells (8). The latter heavily depends on the chemotherapy dose and schedule (74). For example, previous studies have firmly established that cisplatin was not a *bona fide* ICD inducer whereas oxaliplatin is (21, 96, 97). This conclusion was based on studies using super-physiologic dosage and assaying phenotype after a short drug treatment time. For example, Martins et al. studied cisplatin at a dose of IC50, 150 μM, in short-term experiments measuring re-distribution of calreticulin from ER lumen to cell surface within 4-12 hours after drug treatment. They found that cisplatin failed to induce calreticulin cell surface expression, one of the hallmarks of ICD (96). In contrast, Park et al. studied cisplatin at a moderate dose mimicking physiologic conditions, which is dosed at LD40, i.e., 40% of cell death over 3 days, and assayed 1-2 days after drug treatment (98). They found that cisplatin induced immunogenic marker increases including cell surface levels of calreticulin, HSP70, MHC class I, and PD-L1 similar to oxaliplatin in several preclinical models of head and neck cancer. A mouse vaccination assay also confirmed that cisplatin and oxaliplatin induce similar immunogenic changes. Of note, in hepatoblastoma cancer cisplatin also induced tertiary lymphoid structures, which is known to contain both lymphocytes and antigen-presenting cells and correlate with good prognosis (99). Similarly, Zhang et al. found that low-dose gemcitabine treatment (10 nM *in vitro* or 30 mg/kg *in vivo*), rather than high-dose, enhances cell surface exposure of calreticulin in lung cancer and activates NK cells (100).

Apparently, chemotherapy treatment with different dosage and schedule may cause distinct kinetics of DAMPs, including DNA damage responses and/or subsequent cell cycle arrest, which may impact the level of inflammatory signaling, as we will discuss later. These studies suggest that more immunogenic chemotherapy agents can be identified if careful dose and schedule optimization study is carried out. More importantly, it suggests that the efficacy of LDMC can be improved by activating anti-tumor immunity through schedule optimization, rather than only alleviating immune suppression.

## Medium-dose repeating intermittent chemotherapy: optimize schedule to achieve a balance between killing more tumor cells and activating anti-tumor immunity

While low- or moderate-dose chemotherapy has a higher chance to activate anti-tumor immunity by inducing ICD of tumor cells, the concern that low-dosage chemotherapy may not be able to curtail tumor growth remains. One way to address this problem is to optimize the dosing schedule. The total net dosage can be increased by shortening drug free breaks and/or increasing the dose for each injection at preconditions of keeping effective immune activation and no intolerable toxicity to host.

Wu et al. and others found that cyclophosphamide dosed at 140 mg/kg (medium-dose) on every-6-day repeating intermittent chemotherapy (MEDIC) schedule can induce both effective anti-tumor immune responses and persistent tumor regression for 9L rat gliosarcoma, U251 human glioma, and GL261 mouse glioma (10, 32, 91, 101). In contrast, cyclophosphamide dosed on 90 mg/kg on same MEDIC schedule can only induce transient anti-tumor immune responses. Consistently, Manrique et al. found that cyclophosphamide dosage less than 100 mg/kg on an every-7-day repeating schedule was unreliable whereas 100-300 mg/kg dosage on the same schedule was effective in eradicating 4T1 mammary carcinoma, CT26 colon adenocarcinoma, or Panc02 pancreatic ductal adenocarcinoma, when it was combined with toll-like receptor (TLR) agonists (11). These studies suggest that sufficient chemotherapy cytotoxicity against tumor cells is one critic factor that maintains efficient cyclic tumor cell killing and persistent immune cell activation (74).

**Figure 3:**
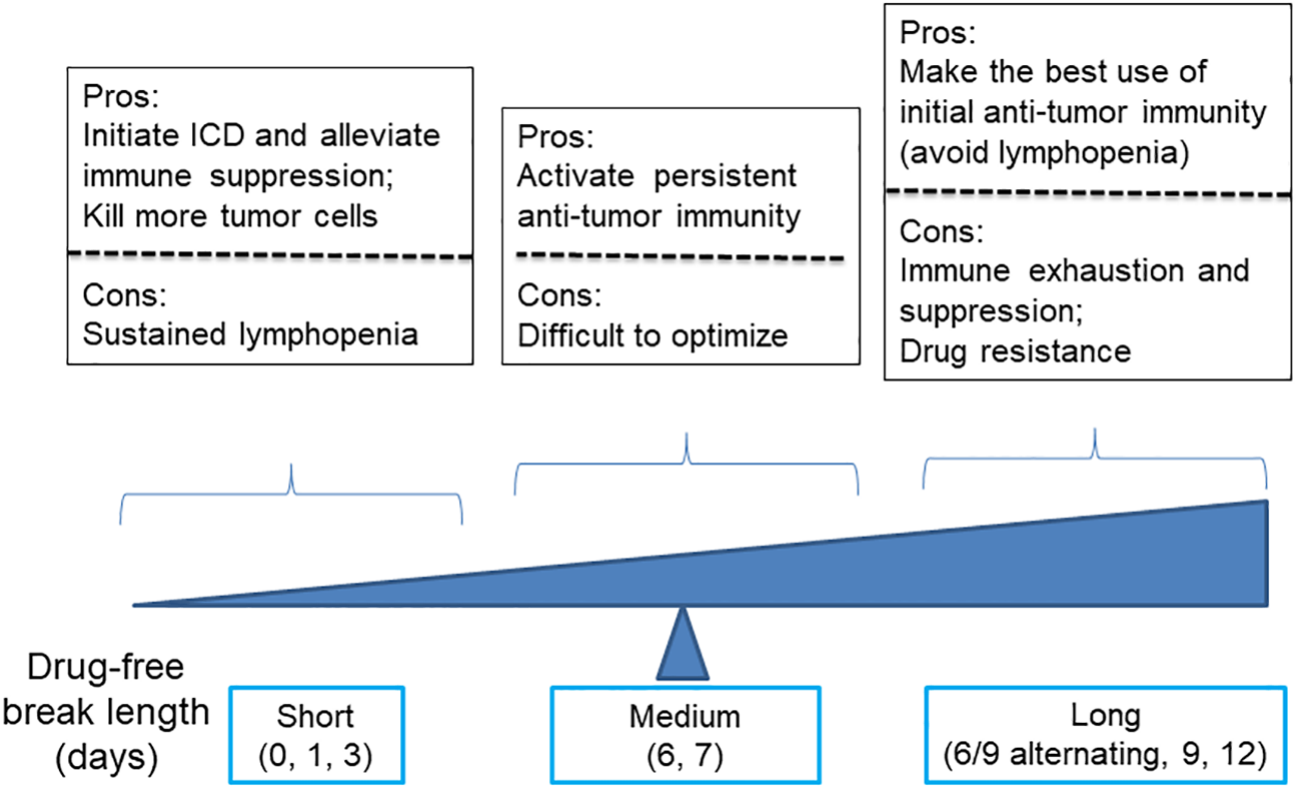
A diagram illustrates the advantages and disadvantages of using cyclophosphamide with varying drug-free intervals to kill tumor cells and stimulate anti-tumor immune responses.

Regarding the dosing schedule, for cyclophosphamide dosed on schedules with drug-free breaks longer than 6 days, such as 6- and 9-day alternating, every 9-day repeating, and every 12-day repeating, they were associated with lower levels anti-tumor immunity and significant tumor relapse, as compared with MEDIC cyclophosphamide treatment (101) (**Figure 3**). Consistently, time course data of gene expression changes indicated that immune suppression followed immune activation in just a few days (10). Therefore, a delicate balance has to be made between the need of extending drug-free breaks to make the best use of chemotherapy-activated immunity and the need of shortening drug-free break to reduce immune exhaustion and/or suppression and drug-resistance developed during long drug-free break (10, 101, 102).

Of note, for cyclophosphamide dosed on a daily or every 3-day repeating schedule, both tumor regression and immune activation were abrogated. Presumably, the failure of anti-tumor immune response activation is combined with compromise of the innate immune system due to strong lymphopenia effects resulting from frequent drug cytotoxicity exposure (91) (**Figure 3**). Consistently, Manrique et al. also found that daily cyclophosphamide of 40 mg/kg failed to activate cyclical immune rebound from strong leukopenia effects and culminate to tumor relapse (11). Thus, another balance needs to be made between the need of increasing dosing frequency to initiate ICD-mediated anti-tumor immunity and kill more tumor cells and the need of extending drug-free break to avoid follow-up chemotherapy-imposed lymphopenia effects (**Figure 3**).

Thus, by using a medium dose given repeatedly on a schedule with medium drug-free break, the dosage of MEDIC schedule is sufficient to kill mass amount of tumor cells without significant toxicity to host (**Table 2**). In the meanwhile, medium drug-free break of MEDIC schedule keeps effective and sustained anti-tumor immunity (**Figure 2D**). Therefore, MEDIC has comparable cytotoxicity to tumor cells as MTD schedule and is superior to MTD and LDMC schedules in activating anti-tumor immunity.

## MEDIC-activated anti-tumor immune response: tumor model dependence and combinatorial regimens for low responsiveness tumor models

Immunogenic chemotherapy activated anti-tumor immune responses is restricted to sensitive tumor models. Wu et al. found that MEDIC cyclophosphamide failed to induce strong anti-tumor immunity and tumor regression in B16F10 tumors and LLC tumors, which have similar *in vitro* sensitivity to cyclophosphamide as GL261 (18). Similarly, Manrique et al. found that MEDIC cyclophosphamide alone cannot induce durable regression in 4T1 breast tumors, CT26 colorectal tumors, Panc02 and KC pancreatic tumors and C57mg breast tumors (11). These findings suggest that there is some intrinsic tumor model difference that determines immune responsiveness upon MEDIC treatment. These less-responsive tumor models may lack some components of DAMP or pattern recognition receptors (PRRs) to initiate anti-tumor immunity (8, 103).

**Figure 4:**
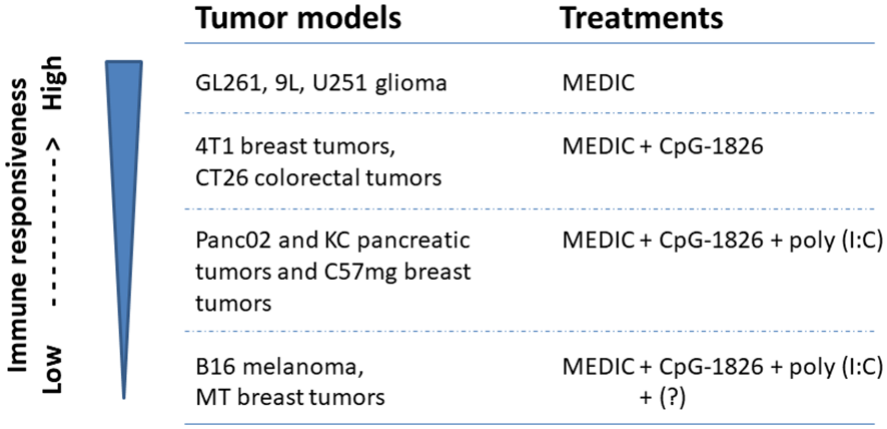
Tumor models with differential immune responsiveness to MEDIC cyclophosphamide treatment. Tumor models that can be durably regressed by MEDIC cyclophosphamide treatment alone or in combination with different TLR agonists in a immune-based mechanisms are shown. "?" stands for an unknown agent(s), combination regimen including poly(I:C), CpG-1826, and cyclophosphamide still cannot eliminate B16 melanoma and MT breast tumors.

Indeed, adding CpG-1826 on day 3 to MEDIC cyclophosphamide regimen durably eradicated 4T1 breast tumors and CT26 colorectal tumors (11) (**Figure 2E**). For the other three tumor models (Panc02 and KC pancreatic tumors and C57mg breast tumors), a TLR3 agonist poly(I:C) has to be added on top of CpG-1826 and cyclophosphamide combination regimen to induce permanent regression (**Figure 2E**). The tumor regression induced by combination regimens requires CD4^+^ and CD8^+^ T cells and tumoricidal myeloid cells, indicative of an immune-based tumor regression mechanism. Thus, there is a gradient responsiveness ranging from the most sensitive GL261, 9L, and U251 glioma, sensitive 4T1 breast tumors and CT26 colorectal tumors, to moderate sensitive Panc02 and KC pancreatic tumors and C57mg breast tumors (10, 11). This exemplifies tumor model-determined differential requirements of TLR agonists to MEDIC cyclophosphamide regimens in order to durably eradicate tumors (**Figure 4**).

However, TLR agonists and cyclophosphamide combination regimen is not a cure for all tumors either. For B16 melanoma and MT breast tumors, even triple combination regimen including poly(I:C), CpG-1826, and cyclophosphamide still cannot eliminate tumors (11) (**Figure 4**). Changing the administration routes of agonists from i.p. to intra-tumor might reverse cold B16 tumor micro-environment, favoring activation of tumoricidal myeloid cells and thus improve the treatment efficacy (104). However, in some cases host or cancer cell gene variants may intrinsically affect the mounting of anti-tumor immune responses, which request different combination therapy regimens (**Figures 2E**, **4**). For example, the loss-of-function variants of TLR4 (receptor for HGMB1) (105) or FPR1 (receptor for ANXNA1) (103) abrogate immune reaction. More defects and corresponding correction methods have been reviewed elsewhere (8).

## Strategies to combine MEDIC with ICIs

While immunogenic chemotherapy given on a MEDIC schedule have great potential to induce a long-lasting response as we discussed above, it is possible that in practice MEDIC chemotherapy still fail to induce durable tumor regression after schedule optimization and combing with some immunotherapies. Then, one alternative approach is to combine MEDIC with ICIs, which is the most successful immunotherapy agents developed in the past years (80).

**Figure 5:**
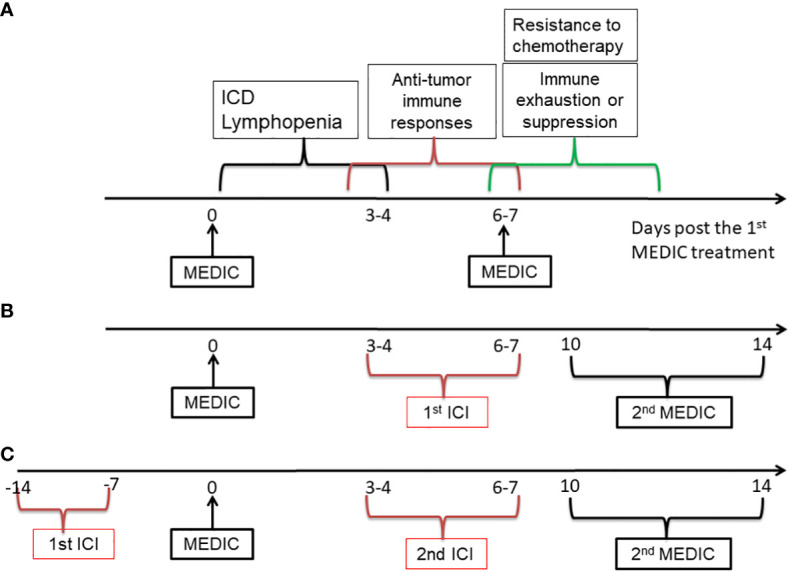
A diagram showing the strategies to combine MEDIC with immunotherapy. MEDIC cyclophosphamide that is carried out on an every 6- or 7-day repeating schedule is used as an example. ICI is used as an example for immunotherapy. **(A)**, Three types of events related to immune responses upon MEDIC treatment is shown on an approximate time scale. **(B)**, A schedule of immunogenic MEDIC followed by ICI is shown. The 2nd ICI following the 2nd MEDIC can be dosed on a similar timing as the 1^st^ ICI. **(C)**, A similar schedule to **(B)**, excepting for using ICI as a precondition agent, when the tumor model have indications of high responsiveness to ICI treatment. The doses of ICI in **(B)** and **(C)** do not have to as high as those used in a 21-day repeating schedule. ICI, immune checkpoint inhibitor.

First of all, it would be ideal to avoid administrating ICI and chemotherapy concomitantly, to reduce strong lymphopenia-mediated abrogation of ICI efficacy. We would propose to administrate ICI at the chemotherapy-free interval. This strategy may take advantage of immunogenic features of chemotherapy to transform “cold” or low responsive tumors into hot. Take MEDIC cyclophosphamide that is carried out on an every 6- or 7-day repeating schedule as an example. Following the 1^st^ MEDIC injection, there will have sequential events such as ICD and lymphopenia approximately from day 0 to day 3, anti-tumor immunity development approximately from day 3 to day 7 beyond, and chemotherapy-resistance and immune exhaustion or suppression development starting roughly from day 6 (**Figure 5A**) (10, 11, 101, 106). It would be ideal to dose ICI approximately from day 3 to day 7 post cyclophosphamide treatments (10, 11, 32, 106) (**Figure 5B**). During this time period immune cells replenishment and anti-tumor immune response starts to mount, which will be followed by various immune suppression events including expansion of PD-L1 expressing tumor cells (10, 107). Thus, ICI dosed at this window may more effectively prevent immune suppression and argument anti-tumor immune response, as compared with dosing ICI and chemotherapy concomitantly. Furthermore, the dose of ICI could be reduced as compared to those in 21-day repeating protocol for two reasons. One is to reduce the toxicity to host. The other is that ICI here is only to prolong the anti-tumor immunity by dampening immune suppression signals for relatively a short period. Following ICI injection, the second chemotherapy injection that aims to kill more tumor cells using chemical cytotoxicity and that will induce lymphopenia and re-set anti-tumor immunity is going to be delivered soon anyway. We hypothesize that the 2^nd^ chemotherapy could be delivered from day 10 to day 14 post the 1^st^ chemotherapy injection (**Figure 5B**), depending on the strength of anti-tumor immunity prolonged by the ICI.

Alternatively, if a tumor model of interest has a high level of TILs or PD-L1 expression that indicates a high responsiveness to a corresponding ICI agent, the ICI could be applied prior to chemotherapy (**Figure 5C**). Likewise, the dose of 1st ICI injection does not have to be as high as in that of 21-day repeating protocol, since the following chemotherapy that will re-set immune system through lymphopenia effects will be delivered soon. Nevertheless, in this scenario it needs to administrate the chemotherapy before immune exhaustion or suppression starts to occur. However, too early administration of chemotherapy may have risk of abrogating immune responses activated by ICI. Thus, there is a balance need to make between utilizing anti-tumor immunity activated by ICI and killing more tumor cells based on chemical cytotoxicity. We estimate that the 1^st^ chemotherapy can be delivered around one or two weeks after ICI injection, depending on the strength of anti-tumor immunity activated by ICI. Once the chemotherapy is injected, the alternating dosing pattern between ICI and chemotherapy can follow previous pattern as shown in **Figure 5B**.

## Cell cycle checkpoint and DNA damage repair pathway impacts genotoxicity-induced immune activation

Regardless of the immunotherapy used in combination with MEDIC, it is crucial to investigate the molecular mechanisms that lead to low responsiveness to MEDIC cyclophosphamide treatment, such as in B16 melanoma and MT breast tumors. Recent studies suggest that investigating DNA damage level or cell cycle progression status after chemotherapy treatment may reveal the immune responsiveness upon chemotherapy treatment and help optimize the chemotherapy schedule (16, 17).

DNA damage response (DDR) is a conserved pathway that maintains genome fidelity. Once cells have experienced DNA damaging events, such as chemotherapy treatment, pathogen infection, or DNA replication error, a set of DDR cascades will quickly initiate to sense DNA damage, repair DNA damage, and/or arrest cell cycle progression if required (108–111). Failed DNA damage repair may eventually lead to increased DNA mutation load, tumorigenesis, inflammation, or cell death (108, 110, 112, 113). Even though DNA released from cell lysis, as one of the DAMP molecules, is a known trigger of immune responses (24, 110, 114), how DDR affects immune response is not fully understood.

**Figure 6:**
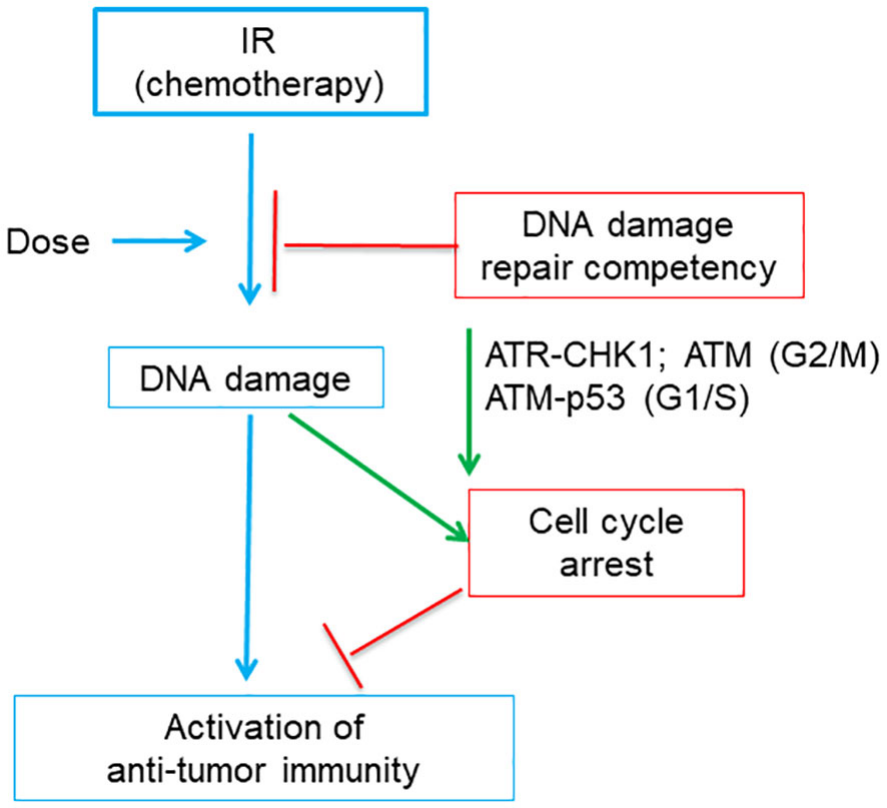
A diagram shows that tumor DNA damage repair competency and related cell cycle checkpoints affects the activation of anti-tumor immunity induced by immunogenic IR or chemotherapy. Tumor DNA damage level is positively correlated with the dose of IR (or chemotherapy). The damaged DNA will be partially repaired by DNA repair pathway and thus the DNA damage level of tumor cells is negatively affected by DNA damage repair competency. Failed DNA damage repair or very high dose of IR may produce excessive DNA damage, which cause cell cycle arrest. Cell cycle arrest is regulated by part of DNA damage response, which includes ATR, CHK1 and ATM for G2/M arrest and ATM and p53 for G1/S arrest, respectively. While persistent cell cycle arrest blocks the activation of anti-tumor immunity, abrogation of cell cycle arrest favors immunity activation.

Recent studies indicated that cell cycle checkpoint and DNA damage repair pathways both impact genotoxicity-induced immune activation (16, 17) (**Figure 6**). IR treatment can induce IFN pathway-based anti-tumor immune responses, which is indispensable for therapeutic efficacy of IR (115). Harding et al. found that for cells undergoing double strand DNA breaks cell cycle progression through mitosis is required to form micronuclei, which further activate IFN signaling through the cGAS-STING pathway (17). This reveals the molecular mechanisms underlying the delayed onset of immune response as compared to DDR at minute-to-hour time scale after genotoxic stress.

Studies on IR further illustrated that dose-dependence of genotoxic stress-mediated IFN pathway activation was related to cell cycle checkpoints (16) (**Figure 6**). The 20-Gy treatment induced excessive DNA damage which arrested MCF10A cells at G2/M phase and failed to induce IFN inflammatory signaling. Abrogation of G2/M checkpoint by inhibitors of ATR or CHK1 restored IFN signaling. In contrast, the 10-Gy treatment that induced less excessive DNA damage and did not induce prolonged G2/M arrest can still generate significant IFN responses. This is consistent with the dose-dependence of chemotherapy-activated immunity in that low- or moderate-dose chemotherapy has a higher chance to induce immunogenic changes than high dose (74, 98).

Furthermore, the IR dose capable of activating immune responses is affected by DNA damage repair pathways (16). DNA damage levels induced by IR will be reduced by the canonical non-homologous end joining (c-NHEJ) pathway. In MCF10A cells deficient of the c-NHEJ pathway, the 10-Gy treatment will accumulate higher DNA damage level than in wild type cells, triggering prolonged G2/M arrest like the 20-Gy treatment did in wild type MCF10A cells, and thus fail to induce immune responses. Abrogation of G2 checkpoint did not restore immune response in 10-Gy-treated c-NHEJ-deficient tumor cells, since cells died soon probably due to mitotic catastrophe. Rather, abrogation of both G1 and G2 checkpoints restore anti-tumor immunity. Of note, loss of G1 checkpoint protein p53 will lead to the change of PRR from DNA damage responsive cGAS to RNA-sensing RIG-I, suggesting that p53, an important player in DDR pathway, affects the mounting of anti-tumor immunity. Furthermore, the 2-Gy IR can induce a higher level of IFN response than 10-Gy did, because the 2-Gy IR only induced a moderate DNA damage level which did not cause G2/M arrest. Thus, the appropriate IR dose able to effectively activate anti-tumor immune responses depends on both cell cycle checkpoints and DNA damage repair competency. Consistently, inhibitors targeting DDR components can greatly increase the efficacy of IR treatment by activating immune responses, and some DDR inhibitors have entered into clinical trials (116–118).

Considering the similarity between IR and chemotherapy treatment-induced genotoxic stress, we think that the biological principles obtained from IR-based study can be largely applied to chemotherapy. Indeed, the biomarkers or signature of DNA repair pathway are associated with the efficacy of both IR and chemotherapy treatment in various cancers (119–122). For example, DNA damage response pathway-altered biliary tract cancers exhibited favorable chemotherapy responses (123). In the stage 4 non-small cell lung cancer (NSCLC), pathogenic variants in DDR pathway genes was associated with higher efficacy of radiotherapy and ICIs (124). The efficacy of chemotherapy can be reduced by STING through suppressing DDR and genotoxicity and DNA instability (125). Of note, there are different DNA damage repair pathways, targeting DNA base damage, base mispairs, small loops, DNA strand breaks, and/or inter-strand crosslinks. Furthermore, heterogeneous defects in DNA damage repair pathways exist in tumor cells (126). For each cancer of interest, it warrants further study how greatly the defect of one particular relevant DNA damage repair pathway would affect the chemotherapy-induced DNA damage level and the optimal chemotherapy dosage.

## Biomarkers indicating immune responsiveness to chemotherapy and/or immunotherapy

Given the inherent molecular heterogeneity across patients and cancer types, one key question remains is how to identify the immune responsiveness in a convenient way (127). Biomarkers indicating immune responsiveness can aid to decide which therapy regimen to take in the beginning and whether there is a need to change therapy protocols during the treatment. By stratifying patients through biomarkers, chemotherapy benefits can be maximized with minimized toxicity side effects and reduced economic burden.

Based on studies in our group and others, tumor basal immune level often correlates with immune responsiveness to chemotherapy (74, 128). But in some cases, tumor infiltrated lymphocytes (TILs) are associated with distinct survival rate after neoadjuvant chemotherapy, e.g. better survival in HER2-positive breast cancer and triple-negative breast cancer (TNBC), but worse survival in luminal–HER2-negative breast cancer (129), (**Table 3**). In other cases such as TNBC with residual tumors, though associated with low TILs before treatment, an increase of TILs after chemotherapy is still indicative of improved prognosis (130, 131). In contrast, increased TILs characterized by high CD3^+^ T cells and CD68^+^ macrophages post-chemotherapy is associated with worse prognosis (132). These findings suggest differential prognostic value of immunologic infiltrates across cancers, calling for specific biomarkers for each cancer type or subtype and treatment (133).

**Table 3:** Representative tumor- or blood-derived biomarkers that are indicative of responsiveness to chemotherapy alone or combination regimen in clinical studys.

Categories	Biomarkers	Cancer types	Treatment	Reference
TILs	TILs	Better survival in HER2-positive breast cancer and TNBC; Worse survival in luminal–HER2-negative breast cancer	Chemotherapy	129
	Increased total TILs after treatment	Better survival in TNBC with residual tumors	Neoadjuvant chemotherapy	130, 131
	An increased subset of TILs (high CD3^+^ T cells and CD68^+^ macrophages) after treatment	Worse survival in TNBC with residual tumors	Neoadjuvant chemotherapy	132
Tumor gene or score	BTK and DPEP2	Lung adenocarcinoma	Immunotherapy, chemotherapy, or radiotherapy	137
	PD-L1 and TILs	Advanced NSCLC	Platinum-based chemotherapy + immunotherapy	138
	Mutation composite scores	NSCLC (stage III & stage IV)	Chemotherapy (pemetrexed + platinum), ICI, or chemotherapy + ICI	139
Blood genes or parameters	BID, FOXP3, KIR3DL1, MAF, PDGFRB, RRAD, SIGLEC1 and TGFB2	Pancreatic ductal adenocarcinoma	FOLFIRINOX	140
	A systemic immune-inflammation index ((neutrophil count × platelet count) / (lymphocyte count))	Gastric or gastroesophageal junction cancer	Neoadjuvant chemotherapy	141
	Total innate lymphoid cells	Metastatic CRC	Chemotherapy (Folfiri, Folfox, or Folfoxiri)	142
	A combined score based on CRP,LDH, and NTL	Not specified	Not specified	143

TNBC, triple-negative breast cancer; Folfiri, 5FU and Irinotecan; Folfox, 5FU and Oxaliplatin; Folfoxiri, 5FU, Oxaliplatin and Irinotecan; FOLFIRINOX, 5-fluorouracil, folinic acid, irinotecan and oxaliplatin; ICI, immune checkpoint inhibitor; TIL, tumor infiltrated lymphocyte.

Along with the application of next generation sequencing, gene profiling of tumor biopsy identifies many biomarkers predicting responsiveness to cancer therapy (123, 134–136). For example, the expression levels of BTK and DPEP2 genes in lung adenocarcinoma are indicative of immune responsiveness and the efficacy of immunotherapy, chemotherapy, or radiotherapy (137), (**Table 3**). Study on advanced NSCLC suggests that only patients with both high PD-L1 expression and high immune infiltration level could benefit from the first-line treatment of chemotherapy plus immunotherapy (138). The mutation composite scores (MCS) generated from tumor mutation profiles can specifically predict the efficacy of chemotherapy, ICI, or chemotherapy + ICI treatment in NSCLC patients, respectively (139). The prognostic function of MCS is highly treatment-specific and, for immune therapy-treated patients, superior to tumor mutation burden and PD-L1 status.

When tumor biopsy cannot be accessed or does not represent the current status anymore after a period of treatment, an alternative method needs to be considered. One convenient approach is to look for biomarkers in blood parameters (3). The eight immune genes (BID, FOXP3, KIR3DL1, MAF, PDGFRB, RRAD, SIGLEC1 and TGFB2) in blood can serve as early circulating biomarker predicting response to FOLFIRINOX (5-fluorouracil, folinic acid, irinotecan and oxaliplatin) after one cycle in patients with pancreatic ductal adenocarcinoma (140), (**Table 3**). This can help decide whether to continue the highly toxic FOLFIRINOX therapy. Immune cell counts in peripheral blood could also be predictive biomarkers. Demircan et al. defined systemic immune-inflammation index (SII) as (neutrophil count × platelet count)/(lymphocyte count). Post-treatment SII value that is lower than pre-treatment was more likely associated with responders in gastric or gastroesophageal junction cancer receiving neoadjuvant chemotherapy (141). Loyon et al. found that increased frequency of total innate lymphoid cells (ILCs), which negatively correlated with anti-tumor responses, is seen in peripheral blood of patients with metastatic CRC after 3-months chemotherapy treatment (142). Seledtsov et al. recommended a combined inflammatory prognostic score based on the blood parameters consisting of C-reactive protein (CRP), lactate dehydrogenase (LDH), and neutrophil-to-lymphocyte (NTL) ratio (143). CRP is a classical marker for acute inflammation, and LDH is an enzyme catalyzing the reversible conversion of lactate to pyruvate. Both CRP and LDH were identified as biomarkers for poor cancer prognosis (144, 145), and increased NTL ratio was often observed in patients with advanced cancers or tumor progression (146). In addition, surrogate tumor responsive markers can also be derived from circulating tumor cells, cell free tumor DNA, and/or RNA in body fluids, such as blood, urine, cerebrospinal fluid, or saliva. These have been extensively reviewed in other literatures (147–149).

As reviewed above, most biomarkers are cancer type- and treatment-specific. Therefore, for a new chemotherapy regimen aiming to eradicate cancers based on anti-tumor immune responses, the available biomarkers for a given cancer type may only be used to stratify preliminary responsive candidates. If a tumor biopsy is available, an ideal approach is to start grouping and stratifying patients based on studies on basal immune infiltrate level, tumor cell cycle checkpoints, and DNA damage repair competency, using either tumor cell culture or patient-derived xenograft models (150). For each group, it warrants to study and optimize the dose and schedule of chemotherapy, and identify more specific biomarkers indicating anti-tumor immune responses.

## Conclusions

We proposed the model of immunogenic chemotherapy-induced long-lasting responses that rely on synergetic interaction between killing tumor cells and inducing anti-tumor immunity. We compared conventional MTD schedule with two types of modified chemotherapy schedules. The LDMC needs to combine with other therapy agents to overcome its low dosage problem. Having comparable dosage as MTD, the MEDIC requires careful optimization to reach optimal dose and schedule to activate persistent anti-tumor immunity. Intrinsic tumor model differences including cell cycle checkpoint integrity and DNA damage repair competency will impact the optimal dose and schedule. Consequently, the MEDIC treatment may also need to combine with immunotherapy to induce a long-lasting response (16, 18, 74). We also proposed strategies to combine immunogenic chemotherapy with immunotherapy, emphasizing the importance of dose and schedule. We believe that efficacy of chemotherapy can be greatly improved either as a mono-therapy or a combination therapy with immunotherapy.

## Author contributions

XH: Writing – review & editing. QR: Writing – review & editing. LY: Writing – review & editing. DC: Writing – review & editing. CM: Writing – review & editing. YZ: Writing – review & editing. JW: Writing – original draft, Writing – review & editing.
